# Getting the most from venous occlusion plethysmography: proposed methods for the analysis of data with a rest/exercise protocol

**DOI:** 10.1186/s13728-015-0027-8

**Published:** 2015-06-09

**Authors:** Stephen Wythe, Thomas Davies, Daniel Martin, Martin Feelisch, Edward Gilbert-Kawai

**Affiliations:** University College London Centre for Altitude Space and Extreme Environment Medicine, UCLH NIHR Biomedical Research Centre, Institute of Sport and Exercise Health, 170 Tottenham Court Road, London, W1T 7HA UK; Integrative Physiology and Critical Illness Group, Clinical and Experimental Sciences, Mailpoint 810, Sir Henry Wellcome Laboratories, Faculty of Medicine, University of Southampton, University Hospital Southampton NHS Foundation Trust, Tremona Road, Southampton, SO16 6YD UK; TamesAide General Hospital, Fountain Street, Ashton-U-Lyne OL6 9RW, Greater Manchester, UK

**Keywords:** Plethysmography, Cell hypoxia, Regional blood flow, Methodology

## Abstract

**Background:**

Venous occlusion plethysmography is a simple yet powerful technique for the non-invasive measurement of blood flow. It has been used extensively in both the experimental and clinical settings. The underlying rationale is that when venous outflow from an extremity is occluded, any immediate increase in volume of this compartment must originate from the on-going arterial inflow. Mercury-in-silastic strain gauges are typically used to measure these volume changes, the rates of which are directly proportional to blood flow.

**Results:**

When using a simple rest/exercise protocol to provide a local or systemic metabolic stimulus to increase blood flow, current methods for analysing the data obtained are often rather simplistic, solely considering the mean increment in blood flow induced by exercise. Previous methodological considerations have focused mainly on issues of reproducibility and accuracy (for instance, by comparing unilateral and/or bilateral measurements) but rarely on what the recorded traces may actually mean.

**Conclusions:**

In this methodological manuscript, we suggest a more detailed approach to processing venous occlusion plethysmography data, one which could provide additional physiological information. Six parameters are described, all of which are easily derived from a simple and reproducible experimental rest/exercise venous occlusion plethysmography protocol.

## Background

Venous occlusion plethysmography (VOP) is a century-old technique used in the non-invasive measurement of blood flow [1]. The underlying rationale is that when venous outflow from an anatomical compartment such as the forearm or leg is occluded, any change in its volume must be due to (and proportional to) continued arterial inflow. This is held to be true in the early phase of occlusion (in the first few seconds): later, exhaustion of available vascular capacitance and limits to tissue compliance may limit both inflow and its associated volume change. Thus, measures of volume change early after venous occlusion are taken to reflect the rate of arterial inflow. Mercury-in-silastic strain gauges are typically used to measure such increases in limb volume.

VOP has been used extensively to study human vascular physiology and the effect of vasoactive drugs such as ACE inhibitors and calcium channel blockers [2, 3]. Additionally, the technique has been used in sport and exercise physiology to study the effects of training regimens [4], dietary supplements [5], and to characterise vascular adaptations in athletes [6], as well as in hypoxic research [7] for the assessment of how high altitude exposure affects peripheral blood flow [8–11]. In all these instances, whilst the underlying principle of VOP remains the same, the exact protocols and analytical methodologies applied somewhat vary. For example, exercise intensity, number of measurements taken and anatomical site (calf vs. forearm) may all vary between protocols.

A repeated measures protocol, with data recorded pre and post a metabolic stimulus, has been used on numerous occasions [4, 6, 9]. Exercise, such as the sequential squeezing of a rubber ring post baseline VOP measurements, has been used for this provocation [9]. The University College London’s Centre of Altitude, Space and Extreme Environment (CASE) Medicine [12] have adapted this simple methodology (further detailed below) and employed it in two studies to date [10, 11]. Data from these studies leads us to conclude that the classical analytical methods often described in the literature [4, 6, 9, 13–17] predominantly focuses on issues relating to the reproducibility and accuracy of VOP measurements but fails to consider what dissimilarities in the recorded traces may mean. In this methodological manuscript, following the description of our VOP protocol and a ‘classic’ method of trace analysis, we suggest six alternative methods of data analysis. Through employing these simple methodological approaches, one may be able to achieve a considerably greater insight into the physiology of, and biological mechanisms behind, peripheral blood flow.

### Venous occlusion plethysmography protocol

The following rest/exercise protocol was utilised by CASE Medicine for their normobaric and hypobaric hypoxic studies. In these studies, the independent variable examined was hypoxic exposure. Ethical approval for the above studies was obtained through the University College London Research and Ethics Committee in accordance with the Helsinki Declaration and informed consent was gained from all participants.

Preceding VOP testing, participant’s dominant forearm circumference and volume were measured. Knowledge of forearm circumference allowed for selection of the correct size mercury-silastic strain gauge (maximum forearm circumference determined by using a tape measure minus 2 cm). Forearm volume measurement allows for the calculation of blood flow per cubic centimetre of tissue and the correction for differences in forearm size and muscle mass. Its measurement is straightforward and takes advantage of Archimedes’ principle: when the arm is slowly immersed into water until the elbow joint, the measured volume of displaced water represents the total forearm volume. This process is then repeated for the dominant hand. The volume of the arm minus the volume of the hand equals the forearm volume. For blood flow measurements, an inflating cuff (SC10D, Hokanson, Bellevue, USA) was placed around the participant’s bicep, to occlude venous blood flow, and connected to a rapid cuff inflator (E20, Hokanson), which was set above venous, but below arterial pressure (50 mmHg). A suitable strain gauge (Hokanson) was placed around the widest part of the forearm and connected to a plethysmograph (EC6, Hokanson). To exclude the hand circulation, which contains a large number of arterio-venous shunts, a segmental pressure cuff (TMC7, Hokanson) was placed around the wrist and inflated to supra-arterial pressure immediately before testing was commenced. Data were recorded on a laptop using the NIVP3 arterial inflow studies software (Hokanson).

Prior to testing, each subject was comfortably seated and allowed to adjust to the environment for 5 min. During this time, the procedure was explained, personal details verified and the software prepared for data capture. So as to prevent the strain gauge from touching the surface of the table during the measurement procedure, their dominant arm was supported by the wrist and elbow with foam pillows. To ensure that the occlusive pressures selected for the dominant arm’s bicep and wrist cuffs would be sufficient to prevent venous outflow and exclude the vasculature of the hand respectively, a baseline blood pressure measurement was taken at rest from participant’s non-dominant arm. On commencing testing, the wrist cuff was manually inflated to 250 mmHg, and the rapid cuff inflator was set to inflate the bicep cuff to 50 mmHg for 7 s at a time. During each 7-s occlusion interval, changes in forearm circumference were detected as changes in electrical resistance in the strain gauge. Five readings were taken at rest, at the end of which the wrist cuff was deflated. A standard 2-min forearm exercise protocol was then performed. This comprised of repeated handgrips of a foam tennis ball to maximum effort using their dominant hand in time with a metronome set at 60 Hz (alternating between squeeze and relaxation every second). Controlling the intensity of exercise in this way is important in ensuring accuracy in subsequent analyses. Once the exercise was completed, the wrist cuff was re-inflated to 250 mmHg and five post-exercise readings were obtained.

### Data extraction

**Fig. 1:**
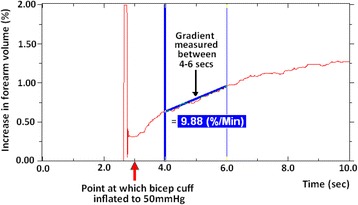
Measuring the trace gradient between 4 and 6 s. The gradient of the VOP trace was measured at two time points, 4–6 and 6–8 s following occlusion. Above illustrates measuring the gradient between 4 and 6 s only, however the same process is used for the 6–8 s time point. It should be noted that the first 4 s on this axis are excluded, as full cuff inflation requires 2.5 s after which a non-specific jump in blood flow is observed. The gradients obtained are an expression of the percentage increase in flow per minute rather than an actual volume of blood over time

Using Hokanson’s proprietary software, the gradient of the five pre-exercise, and five post-exercise traces were measured at two time points: between 4–6 (Fig. 1) and 6–8 s. These timings refer to those on the X-axis of the graph, and it should be noted that the first 4 s on this axis are excluded, as full cuff inflation requires 2.5 s after which a non-specific jump in blood flow is observed relating to the distortion from external compression (movement artefact). As the software measures in straight lines, yet the traces are curved, the two time points are used to give more accurate readings of alterations in flow. The time points may subsequently be compared to give an insight into the dynamics of flow over time, and the flow over 4–8 s may be calculated as the mean of the two measured gradients. Notably, the gradients obtained are an expression of the percentage increase in flow per minute rather than an actual volume of blood over time. Whilst the latter could be calculated using participant’s forearm volume measurements, this may be considered unnecessary given that these values are directly proportional to one another.

## Methods

### ‘Classic’ analysis method

With the gradients obtained, previous studies [9] have calculated the mean value of the five post-exercise measurements, i.e. the ‘mean post-exercise blood flow’. Unfortunately, this method provides no data about i) how participants’ pre- and post-exercise values compare to one another, ii) how individual’s five post-exercise values differ to each other and iii) how such variation differs between individuals. These values may however be important, as we have found considerable variation amongst our data.

### Proposed analysis method

In the following, we present what we believe is an attractive alternative to simple averaging. More specifically, we describe six values that may be derived from the VOP trace gradients, in this instance using the first 2-s time interval (4–6 s).

1. Baseline flow (BF)
  Prior to handgrip exercise, blood flow should not fluctuate as no external stressor has been encountered; thus, calculating the mean value for five consecutive gradients is adequate (Fig. 2). This mean pre-exercise flow reflects forearm blood flow at rest, which we define here as ‘baseline flow’ (BF). In our studies, altered blood flow secondary to differences in either cardiac output, or vascular and/or mitochondrial responses in retort to changes in physical activity, may explain differences in values upon exposure to hypoxia.

**Fig. 2:**
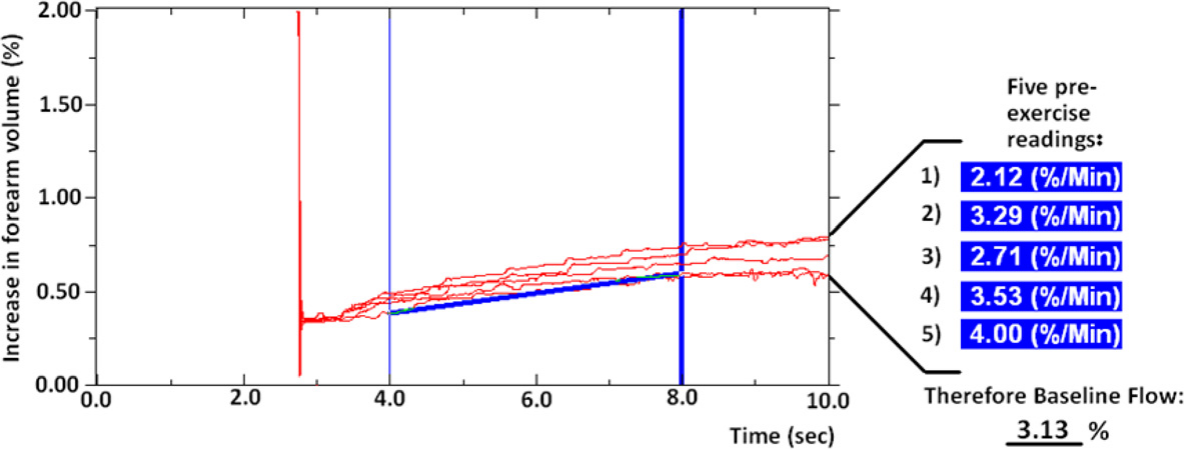
Trend of five pre-exercise readings to demonstrate baseline flow. Five consecutive pre-exercise readings are shown with values taken between 4 and 8 s. Prior to exercise, blood flow should not fluctuate and therefore calculating the mean value of five consecutive gradients reflects forearm blood flow at rest, which we here define as ‘baseline flow’ (BF)
2. Maximal flow response (MFR)
  This is described by the first post-exercise trace gradient. As the name suggests, it gives an indication of maximal blood flow immediately after exercise in response to an increase in oxygen demand secondary to the metabolic stress encountered (Fig. 3). Notwithstanding a small delay between cessation of handgrip exercise, reinflation of the wrist cuff and the start of trace registration, the first recording is indicative of the peak flow response and may be compared to the upstroke of a post-occlusive reactive hyperaemia curve such as that encountered when using near infrared spectroscopy (NIRS) or laser Doppler flowmetry (LDF). In this instance, it may also be of value to look at the area under the curve (AUC) depicted by the trace, as this would provide an indication of the rate at which subjects repay oxygen debt and clear metabolites.

**Fig. 3:**
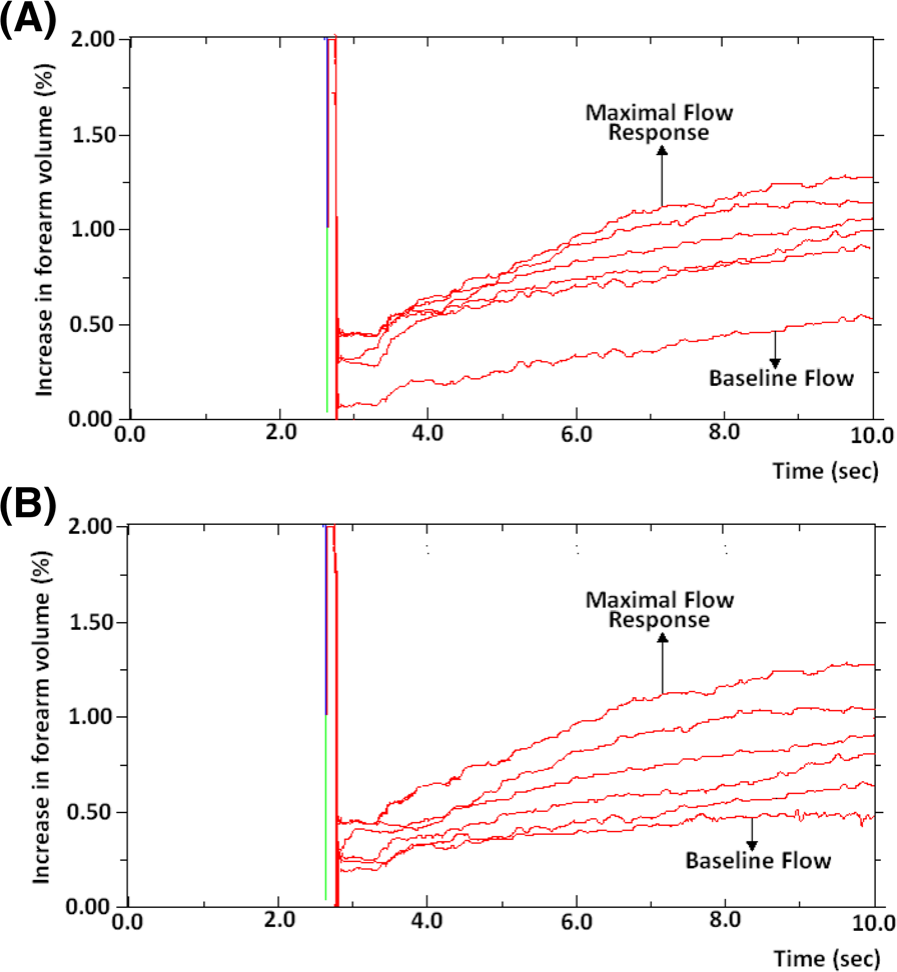
Trend of five consecutive post-exercise readings to demonstrate maximal flow response and differences in subsequent flow. Two examples of five post-exercise readings and baseline flow are seen between 4 and 8 s following occlusion. The first post-exercise trace gradient gives an indication of maximal blood flow immediately after exercise, the maximal flow response (MFR). Individuals demonstrate considerable variation in the rate at which their post-exercise trace gradients return towards baseline. Some remain considerably elevated by the fifth reading **a** whereas others have returned almost to pre-exercise BF values **b**
3. Ratio of maximal flow response to baseline flow
  This value represents the maximal flow stimulation in response to the increased metabolic demand placed on the tissue by a fixed amount of exercise, i.e. the ‘flow reserve’. The higher the ratio, the greater the capacity to increase blood flow in response to exercise.
  Individuals demonstrate considerable variation in the rate at which their post-exercise trace gradients return towards baseline. Some remain considerably elevated by the fifth reading (Fig. 3a) whereas others have returned almost to pre-exercise BF values (Fig. 3b). It is highly likely that these differences between individuals has an underlying biological basis, thus the following methods can provide data on this.
4. Ratio of fifth post-exercise reading to baseline flow
  This reflects the extent to which blood flow has returned to pre-exercise BF values over a fixed time period. It gives an idea of how efficiently metabolites are cleared and whether all oxygen debt has been repaid. Higher ratios would suggest that post-exercise flow has remained high to cope with the metabolic demands whereas lower ratios indicate the debt has been repaid.
5. Ratio of maximal flow response to fifth post-exercise reading
  This allows one to see how quickly blood flow has diminished and returned towards baseline values. A rapid return towards BF would be reflected in higher ratios and suggest that tissues adapt to recover faster under hypoxic conditions. Equally, decreasing ratios would point to a difficulty in repaying oxygen debt and clearing metabolites so flow would remain higher for longer post-exercise. Alternatively, these data may be plotted as a function of time (rather than as a ratio), and thus expressed as the time required for post-exercise flow to return to a set percentage of maximal flow (for example, time needed to return to 50 % of maximal flow (T_50_ [sec]). This can be determined within the NIVP3 software by identifying the trace whose flow rate is closest to half the MFR and then by working out the exact time within that trace that the flow rate reached T_50_ (Fig. 4).

**Fig. 4:**
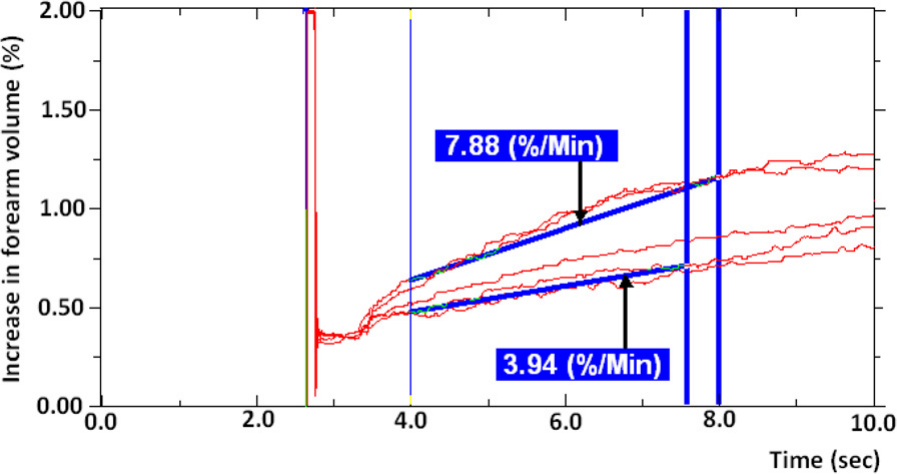
Five post-exercise readings superimposed on one another to illustrate the principal of T_50_. Five post-exercise readings are seen above. T_50_ [sec] is the time needed to return to 50 % of maximal flow. This can be determined within the NIVP3 software by identifying the trace whose flow rate is closest to half the MFR and then by working out the exact time within that trace that the flow rate reached T_50_. In this case, the MFR is 7.88 %/min so T_50_ is found when flow drops to 3.94 %/min. Flow drops to this level around 7.5 s into the fourth reading which makes T_50_ 37.5 s
6. Ratio of maximal flow response to mean post-exercise flow
  This gives an indication of the proportion of recovery that can be attributed to the initial increase in blood flow immediately after exercise. Higher ratios would suggest recovery is due to a large increase in flow which rapidly decreases to baseline rather than a moderate increase in flow over a longer time—something that would be seen with a high mean post-exercise flow value.
  Additionally, further work could be undertaken to assess how normalisation of the responses to forearm volumes may affect any of the studied variables or how variations in the duration of local/whole body exercise/metabolic stress impact VOP responses.

## Summary and outlook

Venous plethysmography has been used for decades to study blood flow in a non-invasive manner. Whilst the data collected yields much useful information when expressed as simple averages, far more information may be extracted using alternative methods of analysis. We describe six values that can easily be derived from the VOP data traces collected using a rest/exercise protocol and discuss their potential significance. These values could improve our understanding of the circulatory response to exercise and hypoxia and, by yielding valuable information regarding the repayment of metabolic/oxygen debt, may provide a window into better understanding oxygen kinetics and mitochondrial function in tissues. Future analysis of VOP traces using a rest/exercise protocol should encompass these methods to maximise one’s insight into the underlying physiology and biological mechanisms they represent.

## Abbreviations

AUC: area under the curve
BF: baseline flow
CASE: University College London’s Centre of Altitude, Space and Extreme Environment Medicine
eNOS: endothelial nitric oxide synthase
LDF: laser Doppler flowmetry
MFR: maximal flow response
NIRS: near infrared spectroscopy
VOP: venous occlusion plethysmography
